# Temperature-dependent increase in the calcium sensitivity and acceleration of activation of ANO6 chloride channel variants

**DOI:** 10.1038/s41598-019-43162-1

**Published:** 2019-04-30

**Authors:** Haiyue Lin, Ikhyun Jun, Joo Han Woo, Min Goo Lee, Sung Joon Kim, Joo Hyun Nam

**Affiliations:** 1 Department of Physiology, Seoul National University College of Medicine, 103 Daehak-ro, Jongno-gu, Seoul 03080 Republic of Korea; 2 The Institute of Vision Research, Department of Ophthalmology, Yonsei University College of Medicine, 50 Yonsei-ro, Seodaemun-gu, Seoul 03722 Republic of Korea; 3 Department of Pharmacology and Brain Korea 21 PLUS Project for Medical Sciences, Yonsei University College of Medicine, 50 Yonsei-ro, Seodaemun-gu, Seoul 03722 Republic of Korea; 4 Department of Physiology, Dongguk University College of Medicine, 123 Dongdae-ro, Gyeongju, 38066 Republic of Korea; 5 Channelopathy Research Center (CRC), Dongguk University College of Medicine, 32 Dongguk-ro, Ilsan Dong-gu, Goyang, Gyeonggi-do 10326 Republic of Korea

**Keywords:** Patch clamp, Ion channel signalling

## Abstract

Anoctamin-6 (ANO6) belongs to a family of calcium (Ca^2+^)-activated chloride channels (CaCCs), with three splicing variants (V1, V2, and V5) showing plasma membrane expression. Unlike other CaCCs, ANO6 requires a non-physiological intracellular free calcium concentration ([Ca^2+^]_i_ > 1 μM) and several minutes for full activation under a whole-cell patch clamp. Therefore, its physiological role as an ion channel is uncertain and it is more commonly considered a Ca^2+^-dependent phospholipid scramblase. Here, we demonstrate that physiological temperature (37 °C) increases ANO6 Ca^2+^ sensitivity under a whole-cell patch clamp; V1 was activated by 1 μM [Ca^2+^]_i_, whereas V2 and V5 were activated by 300 nM [Ca^2+^]_i_. Increasing the temperature to 42 °C led to activation of all ANO6 variants by 100 nM [Ca^2+^]_i_. The delay t_i_me for activation of the three variants was significantly shortened at 37 °C. Notably, the temperature-dependent Ca^2+^-sensitisation of ANO6 became insignificant under inside-out patch clamp, suggesting critical roles of unknown cytosolic factors. Unlike channel activity, 27 °C but not 37 °C (physiological temperature) induced the scramblase activity of ANO6 at submicromolar [Ca^2+^]_i_ (300 nM), irrespective of variant type. Our results reveal a physiological ion conducting property of ANO6 at 37 °C and suggest that ANO6 channel function acts separately from its scramblase activity.

## Introduction

The TMEM16 family of proteins, also known as anoctamins (ANOs), comprise several members that function as calcium (Ca^2+^)-activated chloride channels (CaCCs)^1^. The most well-known CaCCs in this group, TMEM16A (ANO1) and TMEM16B (ANO2), play fundamental roles in various physiological processes. ANO1 mediates transepithelial secretions in several gland tissues, possesses pacemaker potential for gastrointestinal motility, and acts as a heat sensor in nociception^2–6^, whereas ANO2 is believed to be associated with olfactory sensory transduction and photoreceptor signalling^7,8^.

In contrast, ANO6 (TMEM16F) has a dual function, acting as a phospholipid scramblase as well as a CaCC. As a scramblase, ANO6 plays essential roles in platelet coagulant activity, induces pro-apoptotic signals in lymphocytes, induces phagocytosis as a downstream target of P2X7 receptors in macrophages, deposits hydroxyapatite in osteoblasts, and restricts T cell responses to maintain the balance of the immune reaction^9–16^. As an ion channel, ANO6 shows voltage-dependent activity and has been suggested to be a component of the outwardly rectifying chloride channel^17,18^, even though there is a suggestion that it is a Ca^2+^-activated cation channel^19^. However, the role of ANO6 as a physiologically meaningful ion channel remains controversial. This debate largely stems from the fact that ANO6 activation requires a very high intracellular Ca^2+^ concentration ([Ca^2+^]_i_). The reported half-maximal Ca^2+^ concentrations (EC_50_) for ANO6 are quite variable, ranging from several to 106 μM^20–22^. Furthermore, even with a high [Ca^2+^]_i_, appearance of the ANO6 current is generally delayed and requires several minutes for activation. These properties are largely different from those of the ANO1 current that is activated with a submicromolar EC_50_ (~400 nM) of [Ca^2+^]_i,_ showing no delayed activation in response to Ca^2+^ signals^23,24^. Because of the relatively low Ca^2+^ sensitivity and delayed activation properties of ANO6, unlike ANO1, which is activated directly by ER calcium release during G protein coupled receptor (GPCR) stimulation, ANO6 might be activated by sustained calcium influx through store-operated calcium entry after GPCR stimulation^25^.

Although there are several alternative splicing variants of ANO6, most previous studies have dealt with variant type 1 (V1). According to a recent study, three types (V1, V2, and V5) of ANO6 show CaCC activity with differential Ca^2+^ sensitivity and time delay for peak current generation: V2 showed a shorter delayed time than V1 and V5, whereas V2 and V5 showed a higher Ca^2+^ sensitivity than V1. However, even the relatively more sensitive variants of ANO6 still required several micromolar [Ca^2+^]_i_ and >100 s delayed time for significant activation to be detected^26^.

Experimental temperature generally affects the activities of ion channels by increasing the maximum current amplitudes and kinetics of activation/inactivation^27,28^. In response to a temperature change, ANO1 showed increased activated current densities, with a Q_10_ (the 10 °C temperature coefficient) value of approximately 19.4, and an increase in the [Ca^2+^]_i_ lowered the temperature threshold for ANO1 activation^5^. Based on this background, we hypothesised that the low Ca^2+^ sensitivity and the delayed activation of ANO6 might be due to the experimental temperature (i.e., room temperature) applied in previous studies. Therefore, in this study, we investigated the effects of physiological temperature (i.e., 37 °C) on the activity of ANO6 variants in terms of their Ca^2+^ sensitivity and delayed activation. We found that although each variant differed, all variants could be activated at temperatures between 37 and 42 °C under submicromolar Ca^2+^ concentrations with short delays. In contrast, the [Ca^2+^]_i_-dependent scramblase activity of ANO6 was contrary altered by temperature. This work is expected to clarify the role of ANO6 as a chloride channel under physiological conditions beyond its known function as a scramblase.

## Results

### Membrane expression and ion channel activity of ANO6 variants

**Figure 1:**
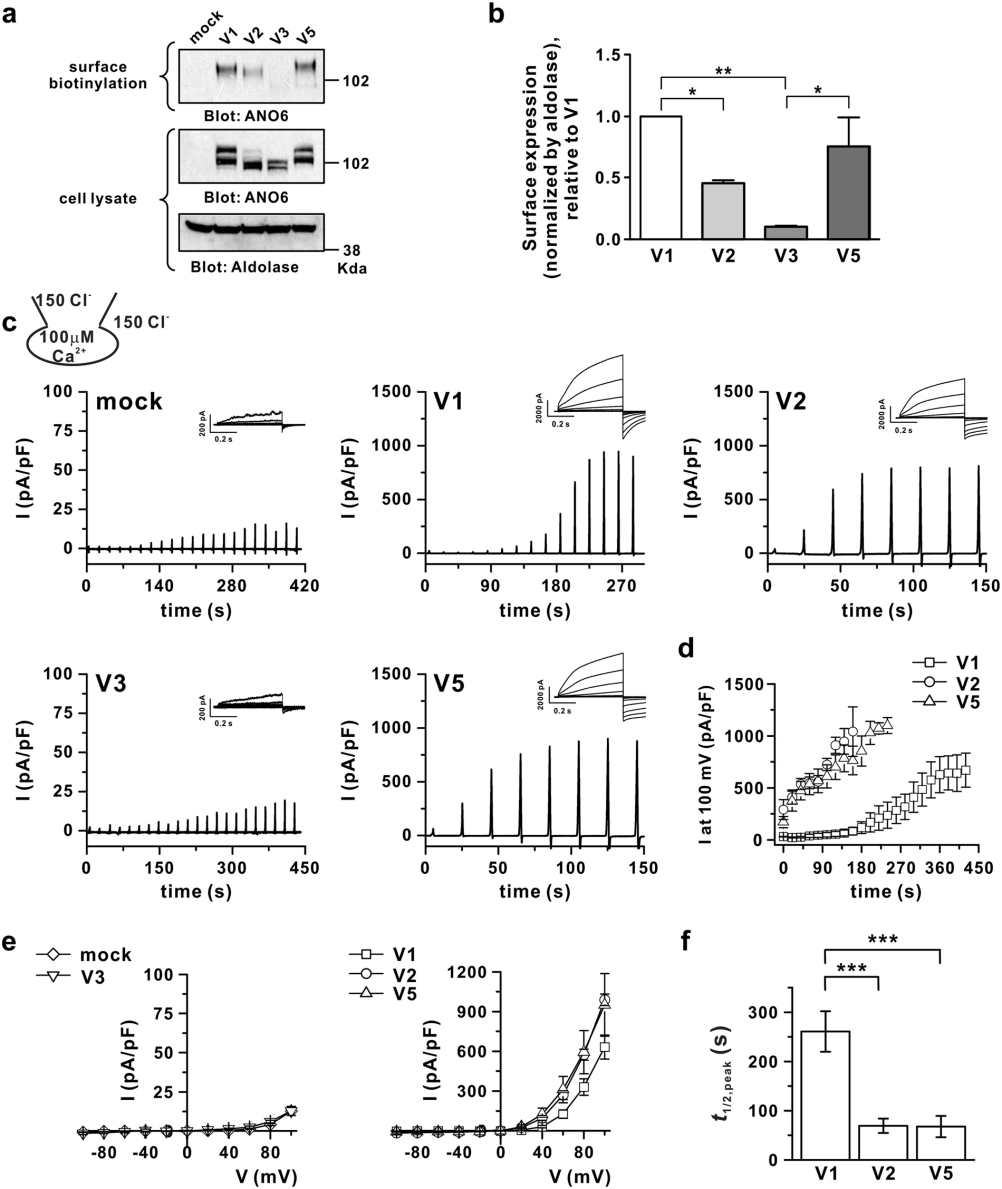
Membrane expression and currents of hANO6 transcript variant V1, V2, V3, and V5. (**a**) Surface biotinylation assay in HEK293T cells transfected with pcDNA3.1 expressing hANO6 V1, V2, V3, and V5. (**b**) Summary of membrane expression of each variant normalised to V1 (n = 4). *P < 0.05, ***P < 0.001, compared to V1. (**c**) Representative trace recordings of ANO6 currents (I_ANO6_) with whole-cell patch clamping with 100 μM free calcium in pipette solution at 27 °C for the mock (upper left panel), ANO6 variants V1 (upper middle panel), V2 (upper right panel), V3 (lower left panel), and V5 (lower right panel) expressed in HEK293T cells. The relative step pulse trace for each variant is presented in the right upper panel of the ramp pulse chart trace. Under the whole-cell patch clamp, a ramp-like pulse from −100 mV to 100 mV (duration time 3 s) was applied every 20 s (holding voltage, −60 mV) and the step pulse protocol was started from a holding potential of −60 mV and then depolarised for 0.5 s from −100 mV to +100 mV with 20 mV increments. (**d**) The time-based plots of activated outward currents at 100 mV recorded in V1, V2, and V5-transfected HEK293T cells. Time 0 represents the beginning of whole-cell patch recordings. (**e**) The peak corresponding current (I)–voltage (V) relationship curve obtained from mock - diamond and V3 - down-triangle (left-panel), V1- square, V2 - circle, V5 - up-triangle (right-panel). (**f**) Summary bar graph of delay time (*t*_1/2, peak_) of I_ANO6_ generation (time from the start of whole-cell patch recordings to half maximal current activation) detected with 100 μM [Ca^2+^]_i_. Data are presented as the means ± SEM (n = 10 for V1, V2, and V5; n = 4 for V3 and n = 5 for mock). ***P < 0.001 compared to the control. For uncropped blots see Supplementary Fig. S1.

First, we confirmed the membrane localisation of ANO6 variants using a surface biotinylation assay. The results indicated insignificant membrane expression of ANO6 V3 (Fig. 1a). The membrane expression of V1 was higher than that of V2, with an intermediate level of V5 (Fig. 1a,b). The molecular sizes of ANO6 variants in the cytosolic fraction appeared to reflect differential glycosylated states, with the Golgi-mediated complex in its glycosylated form being detected in the surface biotinylation assay (Fig. 1a). To confirm ANO6 variant functional activity, a whole-cell patch clamp assay was conducted for mock (green fluorescent protein (GFP)), V1, V2, V3, and V5 of ANO6-transfected HEK293T cells using symmetrical N-methyl-D-glucamine-Cl (NMDG-Cl) solutions with 100 μM [Ca^2+^]_i_, which is known as the maximal Ca^2+^ concentration for ANO6 variant activation^28^. Ramp-like depolarisation from −100 mV to +100 mV was applied every 20 s after the membrane break-in to attain the whole-cell configuration followed by a step pulse from −100 mV to +100 mV with 20 mV increments after the activated current reached a steady state. As shown in Fig. 1c,d, V1, V2, and V5 transfected into HEK293T cells produced outwardly rectifying chloride current (I_ANO6_) at 100 μM [Ca^2+^]_i_, whereas V3 elicited insignificant membrane currents with the same level as mock. Even in maximal [Ca^2+^]_i_ for ANO6, ANO6 variants showed characteristic delayed activation, with the different time delays to reach half of the peak amplitude (*t*_1/2,peak_) for variants being 260.9 ± 41.2, 69.4 ± 14.5, and 67.8 ± 21.7 s for V1, V2, and V5, respectively (Fig. 1e).

### Increasing the Ca^2+^ sensitivity of ANO6 variants via temperature

**Figure 2:**
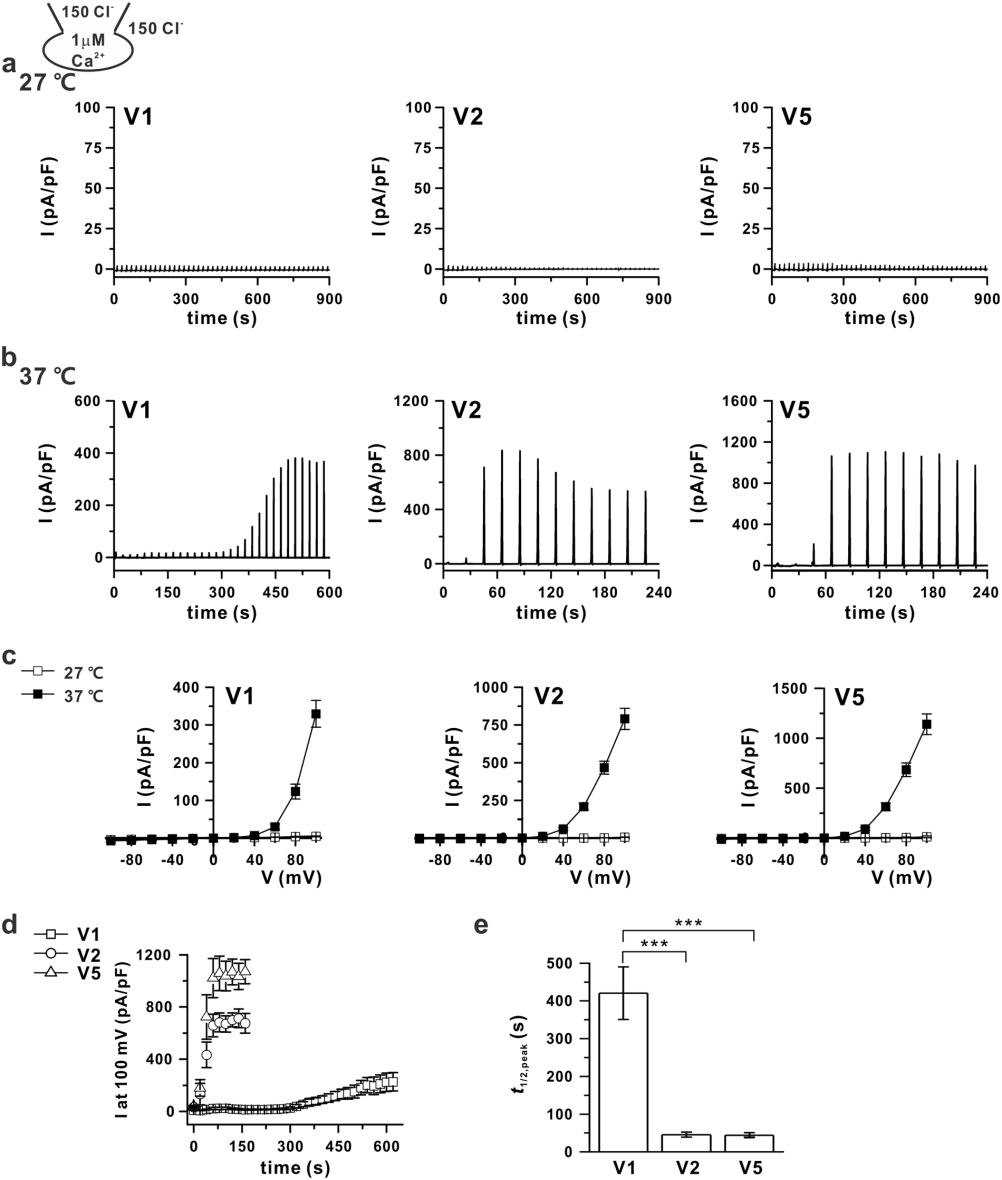
Increased calcium sensitivity of ANO6 variants at 37 °C. (**a**,**b**) Representative traces of ANO6 currents (I_ANO6_) with whole-cell patch clamping with 1 μM free calcium in pipette solution at 27 °C (**a**) and 37 °C (**b**) for the ANO6 variants V1 (left panel), V2 (middle panel), and V5 (right panel) expressed in HEK293T cells. The voltage protocol is already described in Fig. 1. (**c**) I-V relation curve obtained from the peak current of each variant (V1-left panel, V2-middle panel, and V5 -right panel) at 27 °C (open-square) and 37 °C (solid-square). (**d**) The time-based plots of activated outward currents at 100 mV recorded in V1, V2, and V5 transfected HEK293T cells at 37 °C with 1 μM [Ca^2+^]_i_. (**e**) Summary bar graph of the *t*_1/2,peak_ of I_ANO6_ detected at 37 °C. Data are presented as the means ± SEM (n = 13 for V1 and V2, n = 15 for V5). ***P < 0.001 compared to the control.

**Figure 3:**
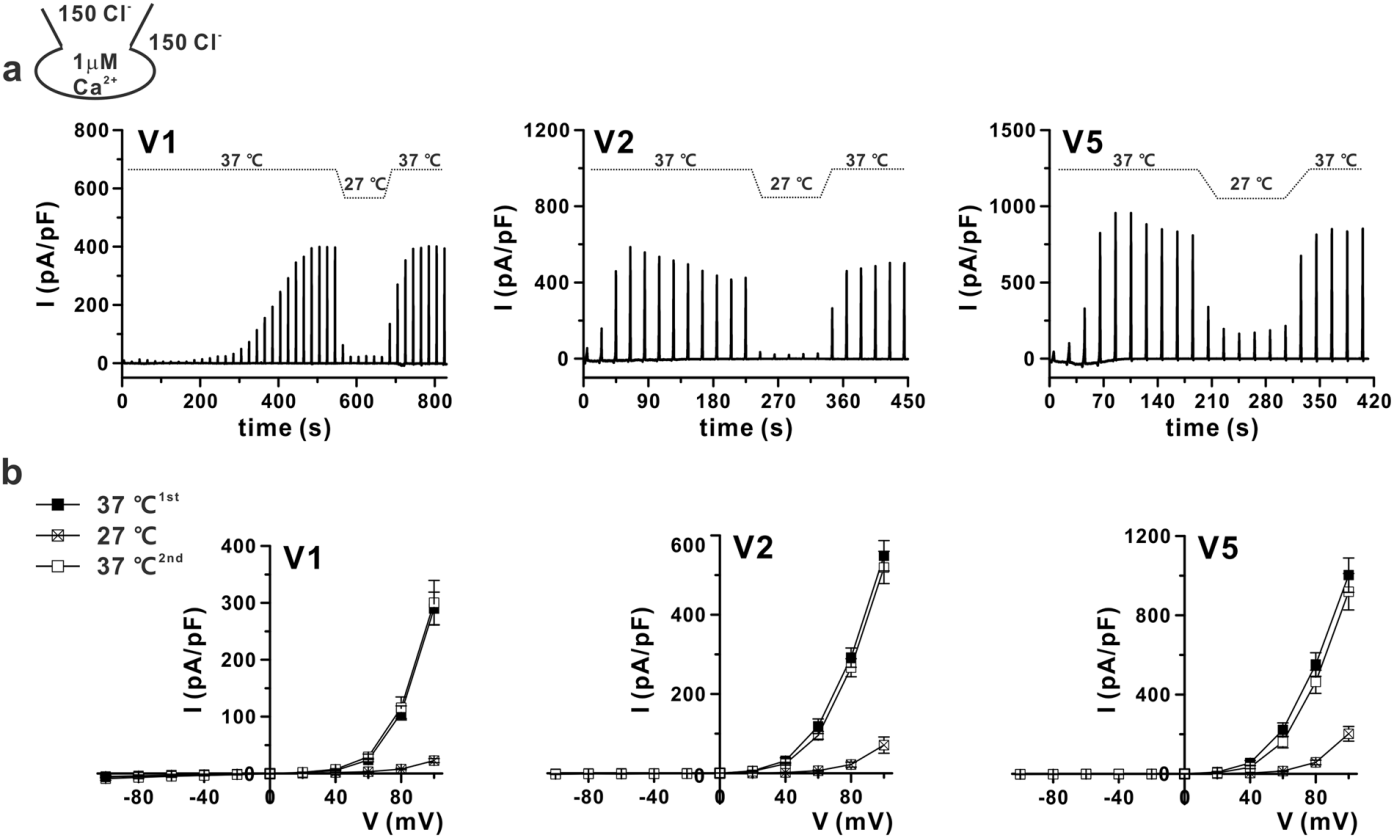
Reversible effect of temperature changes on I_ANO6_. (**a**) Representative current traces of I_ANO6_ of each variant (ANO6 V1 - left panel, V2 - middle panel, and V5 - right panel) expressed in HEK293T cells from 37 °C to 27 °C and returned to 37 °C. (**b**) The peak current (I)–voltage (V) relationship curve of ANO6 variants V1 (left panel), V2 (middle panel), and V5 (right panel) detected at first 37 °C (solid-square), 27 °C (x, centre-square), and second 37 °C (open-square). The same whole-cell patch protocol was used as described in Fig. 2 (n = 8 for V1, n = 6 for V2, and n = 7 for V5).

A whole-cell voltage clamp was conducted for V1, V2, and V5 of ANO6-transfected HEK293T cells with 1 μM [Ca^2+^]_i_. None of the variants was activated by 1 μM [Ca^2+^]_i_ at 27 °C given that no currents were generated, even over long-term (15 min) recordings, as observed by Scudieri *et al*.^24^ (Fig. 2a,c). In contrast, at 37 °C, V1, V2, and V5 generated I_ANO6_ with 1 μM [Ca^2+^]_i_ (Fig. 2b,c). The *t*_1/2,peak_ of I_ANO6_ was significantly longer for V1 than for V2 and V5 (Fig. 2d,e, Table 1). In addition, we conducted an experiment to determine if this temperature effect was reversible. After confirming the full activation of I_ANO6_, changing the bath perfusate to one equilibrated at 27 °C readily decreased the current amplitude, whereas upon subsequent return to 37 °C, the currents increased without delayed activation, activating almost in the same time as the rate of bath temperature exchange in 60–70 s (Fig. 3a). It was notable that the reverse, i.e., the recovery of I_ANO6_, was almost completed (Fig. 3b). One peculiarity is that there is a difference in size between the variants, but once activated at 37 °C, I_ANO6_ is still somewhat active at 27 °C. In particular, V5 showed 30% activity at +100 mV compared to I_ANO6_ at 37 °C.

**Table 1:** *t*_1/2,peak_ (s) of ANO6 variants with different Ca^2+^ concentrations at 27 °C, 37°C, and 42°C (means ± SEM).

[Ca^2+^]_i_	3 μM		1 μM		300 nM		
	27 °C	37 °C	27 °C	37 °C	27 °C	37 °C	42 °C
V1	782.2 ± 76.5	406.9 ± 65	N.R	420.6 ± 69.4	N.R	N.R	236.8 ± 168.5
V2	140.8 ± 39.2	39.52 ± 5.8	N.R	45.6 ± 6.6	N.R	90.5 ± 10.1	76.8 ± 20.3
V5	103.2 ± 18.1	27.7 ± 4.3	N.R	44.2 ± 6.5	N.R	80.8 ± 16.8	49.9 ± 14.5

N.R; No response.

**Figure 4:**
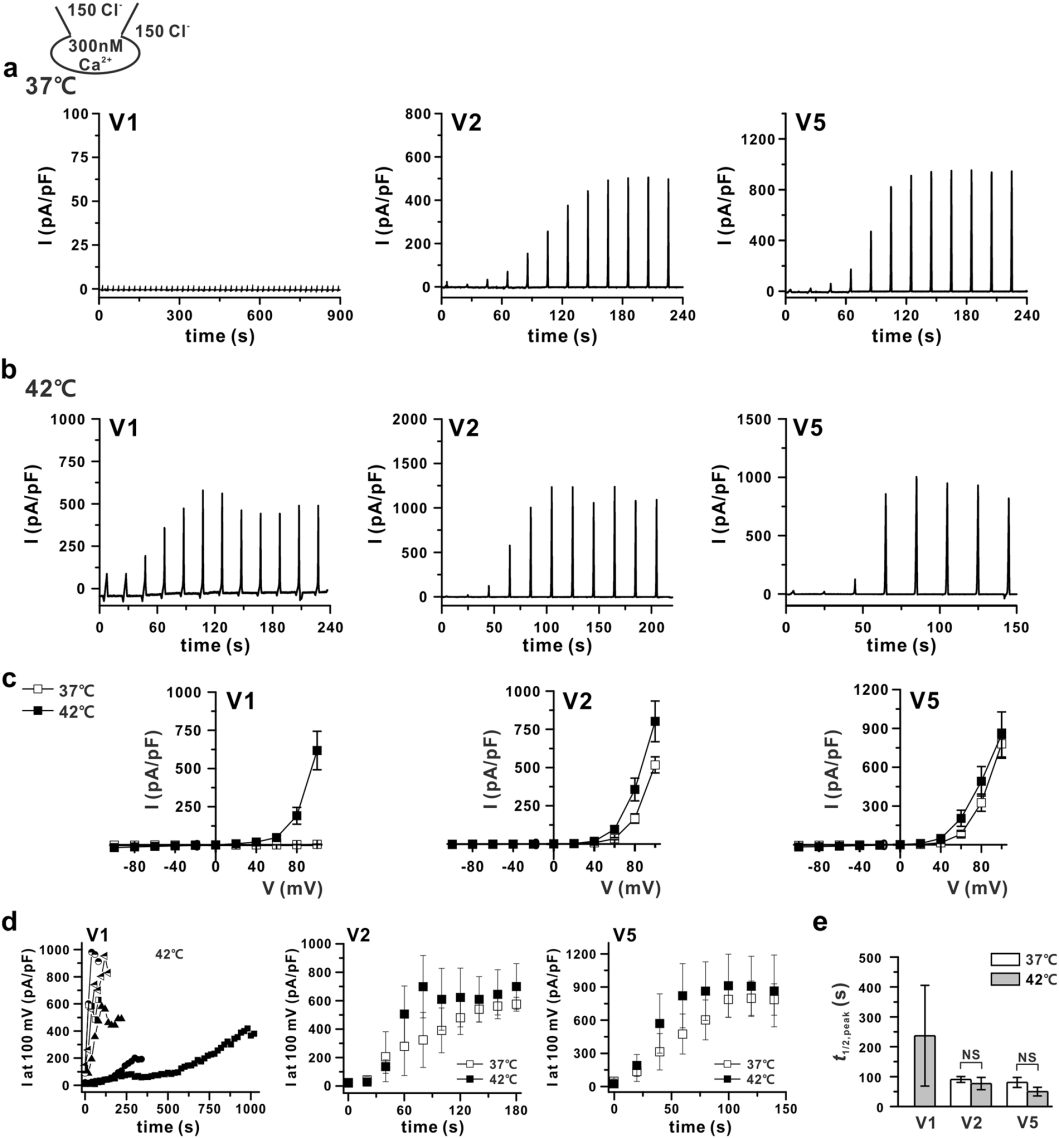
Temperature-dependent activation of ANO6 variants by submicromolar [Ca^2+^]_i_. (**a**,**b**) Representative trace of the I_ANO6_ of V1 (left), V2 (middle), and V5 (right) with 300 nM free calcium in pipette solution at 37 °C (**a**) and 42 °C (**b**) expressed in HEK293T cells. The pulse protocol is the same as that described in Fig. 2. (**c**) I-V relationship curves for the peak amplitude of each variant (V1 - left, V2 – middle, and V5 - right) detected at 37 °C (open-square) and 42 °C (solid-square) with 300 nM [Ca^2+^]_i_. (**d**) The plots of activated outward currents at 100 mV recorded in V1, V2, and V5-transfected HEK293T cells with 300 nM [Ca^2+^]_i_. Right panel shows different cases of V1-induced currents at 42 °C, middle and left panels show V2 and V5-induced currents, respectively, at 37 °C (open-square) and 42 °C (solid-square). (**e**) Summary bar graph of *t*_1/2,peak_ values of V1 detected at 42 °C; V2 and V5 detected at 37 °C and 42 °C. Data are presented as the means ± SEM (n = 4 for V1, n = 7 for V2, and V5 at 37 °C; n = 5 for V1, n = 8 for V2, and n = 7 for V5 at 42 °C).

**Figure 5:**
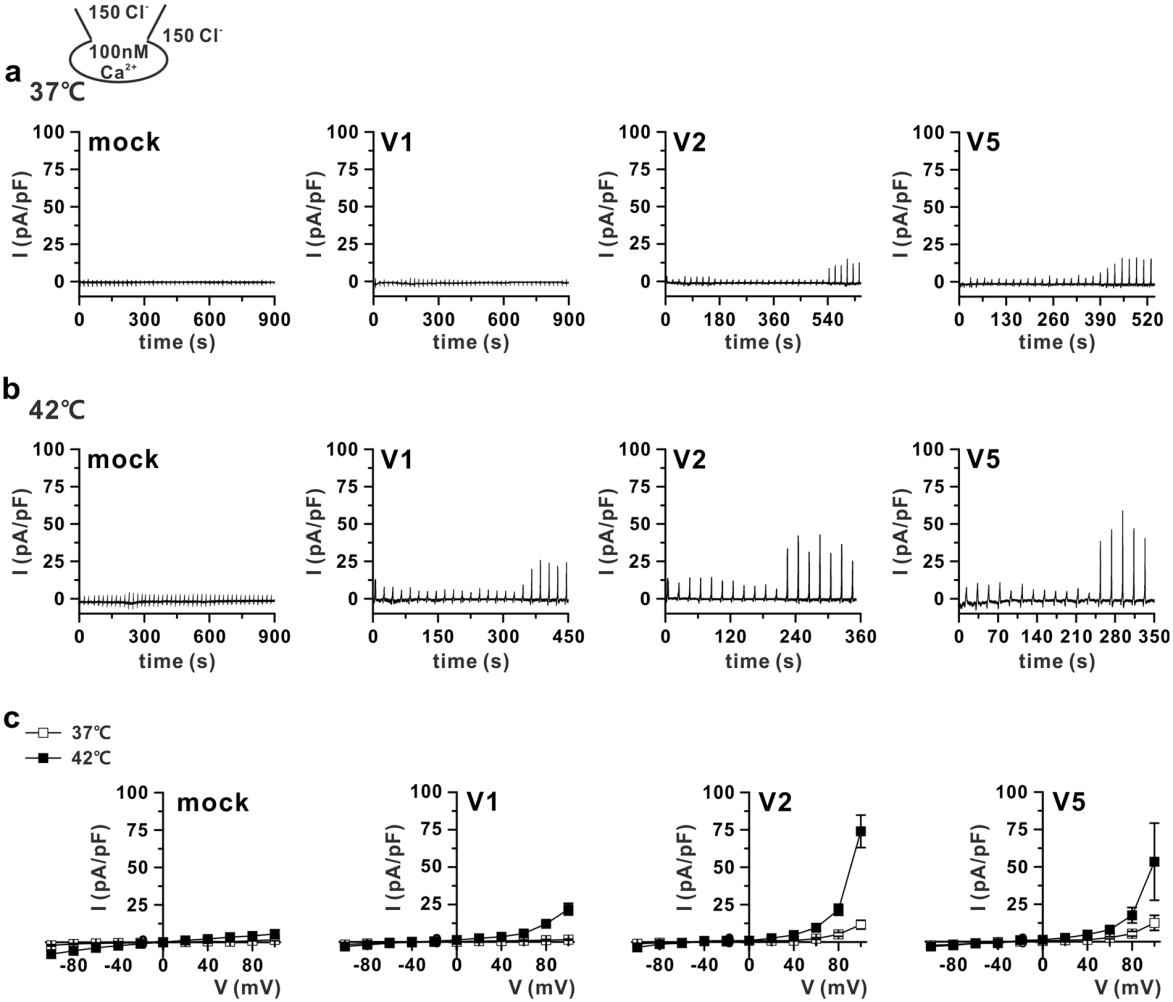
Partial activation of ANO6 variants 1, 2, and 5 by resting [Ca^2+^]_i_ at 42 °C (**a**,**b**) Representative trace of the I_ANO6_ of mock, V1, V2, and V5 at 37 °C (**a**) and 42 °C (**b**) with 100 nM [Ca^2+^]_i_ expressed in HEK293T cells. The pulse protocol is the same as that described in Fig. 2. (**c**) The peak I-V relationship curves obtained from mock and each variant detected at 37 °C (open-square) and 42 °C (solid-square) (n = 5 for all variants at 37 °C; n = 4 for V1, n = 7 for V2 and V5 at 42 °C).

Although the ANO6 variants could be activated even with 1 μM [Ca^2+^]_i_, the initial appearance of I_ANO6_ and steady-state activation took a considerable amount of time; such abnormally long sustained high calcium conditions would occur only under pathological conditions. Thus, we next asked whether raising the temperature to physiological values would induce I_ANO6_ at the submicromolar range of [Ca^2+^]_i_ in HEK293T cells expressing V1, V2, or V5. In particular, with 300 nM of [Ca^2+^]_i_ at 37 °C, V2 and V5 showed significant I_ANO6_ activation. However, V1-expressing cells did not show any current activation up to 900 s after the membrane break-in (Fig. 4a,c, Table 1). To identify whether an increased temperature could activate V1, we raised the temperature to 42 °C. As shown in Fig. 4b, increasing the temperature to 42 °C could activate all ANO6 variants. However, the *t*_1/2,peak_ was not significantly decreased (Fig. 4d,e). In cases of V1, activation at 42 °C appeared to have already occurred for the first ramp (Figs 4b and S2a), showing a linear I-V relationship (Supplementary Fig. S2b), which was different from the completely activated outwardly rectifying ANO6 current (I_ANO6_) (Supplementary Fig. S2c). The weak outwardly rectifying anionic current was activated in the initial period after membrane break-in. This conductance was supposedly an activation of volume-regulated anion current due to putative osmotic imbalance in the initial period of intracellular dialysis. Therefore, we did not consider the initial semi-linear current as I_ANO6_. Interestingly, when the [Ca^2+^]_i_ was further lowered to 100 nM, close to the resting [Ca^2+^]_i_, ANO6 variants V2 and V5 generated very small and partially activated outward currents at 37 °C (11–15 pA/pF at 100 mV), whereas V1 did not (Fig. 5a,c); at 42 °C, V1 showed slightly activated outward currents when V2 and V5 still generated small but significant outward rectifying currents, as the currents elicited at 100 mV I_ANO6_ were approximately 21.7 ± 3.9, 73.9 ± 10.8, and 53.4 ± 25.7 pA/pF for V1, V2, and V5, respectively (Fig. 5b,c). However, all ANO6 variants did not generate any current in a calcium-free pipette solution even at 42 °C (see Supplementary Fig. S3).

### Comparison of *t*_1/2,peak_ of ANO6 variants at 3 µM

**Figure 6:**
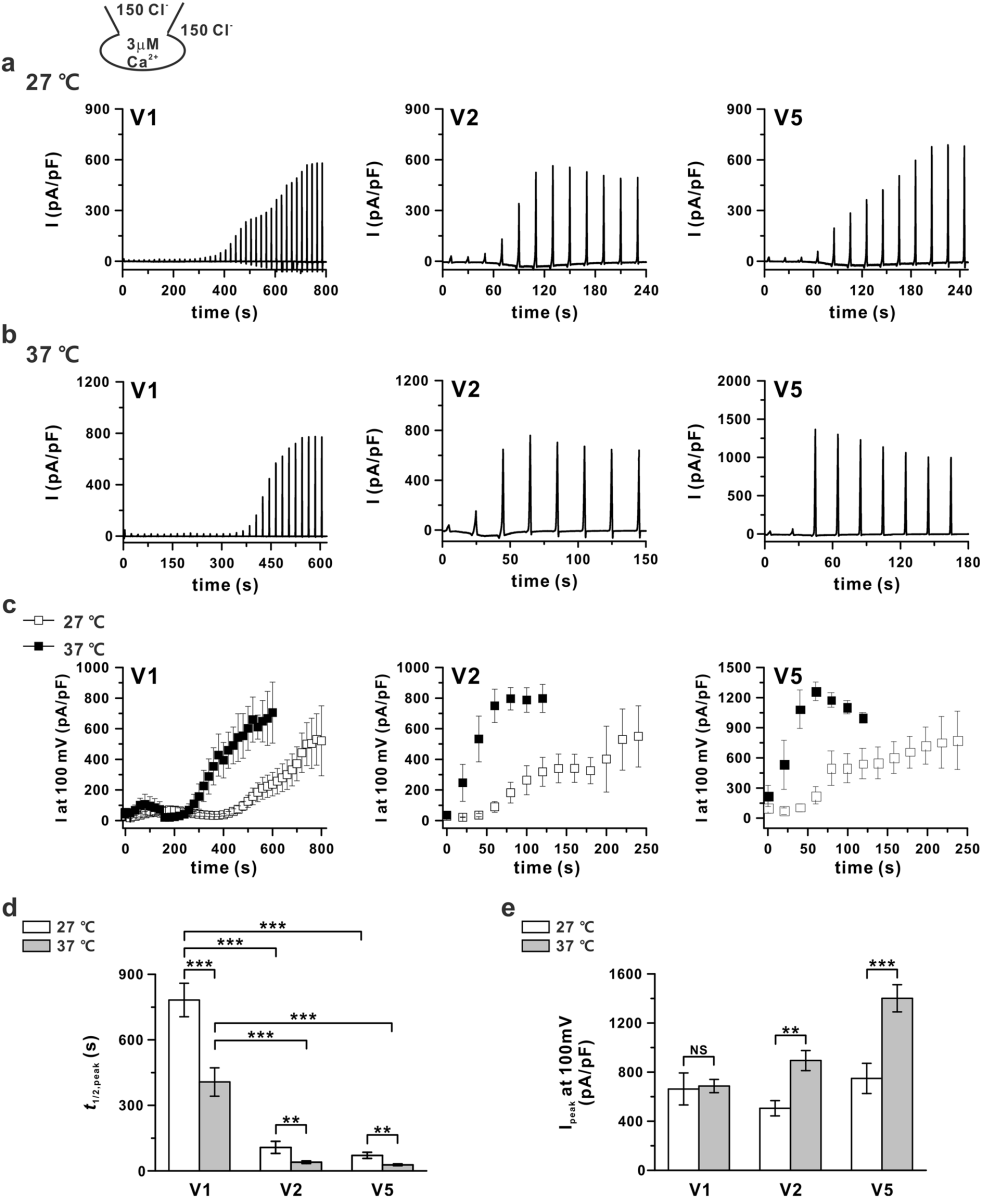
Accelerated activation of I_ANO6_ at 37 °C. (**a**,**b**) Representative current traces of I_ANO6_ generated from three variants (V1, left panel; V2, middle panel; and V5, right panel) with 3 μM free calcium in pipette solution at 27 °C (**a**) and 37 °C (**b**) in ANO6 variant-overexpressing HEK293T cells. The whole-cell patch recording pulse protocol was the same as that described in Fig. 2. (**c**) Time-based activated outward currents at 100 mV obtained from each variant detected at 27 °C (open-square) and 37 °C (solid-square). (**d**) Summary bar graph of *t*_1/2,peak_ of I_ANO6_ of each variant estimated from the trace chart. (**e**) Peak currents elicited by 3 µM [Ca^2+^]_i_ at +100 mV holding potential from each variant at 27 °C (white bar) and 37 °C (grey bar). Data represent the means ± SEM (n = 10 for 27 °C and n = 15 for 37 °C, for all variants). NS indicates not significant, **P < 0.01, ***P < 0.001 compared to the control.

To compare the change in *t*_1/2,peak_ according to the temperature change under the condition of I_ANO6_ activation at 27 °C, [Ca^2+^]_i_ was fixed at 3 μM and whole-cell patch clamp was conducted. In all three variants, I_ANO6_ reached the peak amplitude faster at 37 °C than at 27 °C (Fig. 6a–d, Table 1). Among the variants, the activation speed of V1 was generally slower than that of V2 and V5 at both temperatures (Fig. 6d), whereas the *t*_1/2,peak_ was reduced to approximately 40–50% by increasing the temperature from 27 °C to 37 °C in all variants. However, in case of the peak current generation, V1 showed almost no change in peak current amplitude due to temperature change, and only V2 and V5 showed a statistically significant increase in peak current amplitude, an approximately 1.7- and 1.8-fold increase, respectively (Fig. 6e).

### Differential properties of ANO6 variants in the excised patch of the membrane

**Figure 7:**
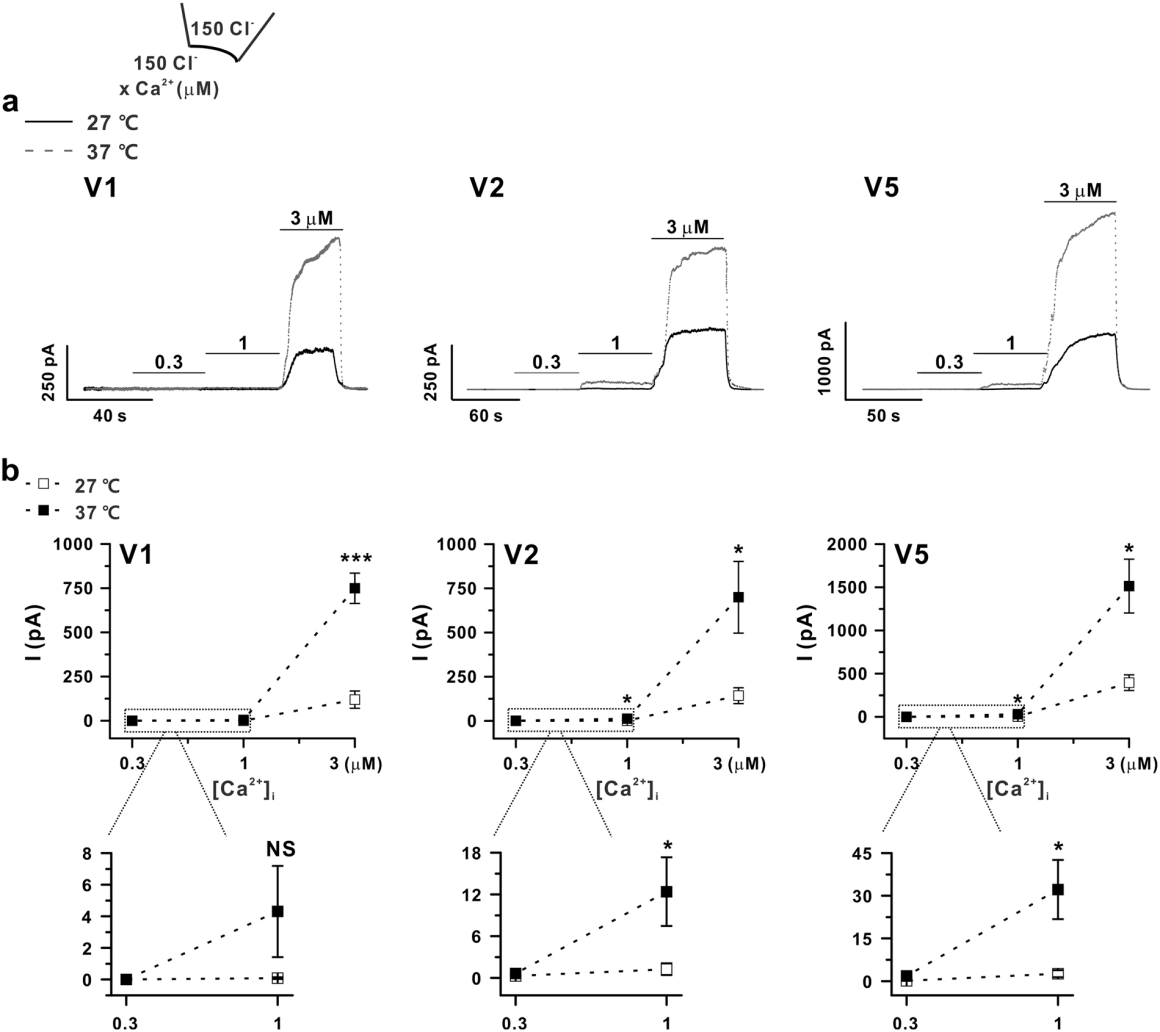
Calcium sensitivity and activation kinetics of ANO6 in excised patches. (**a**) Representative macroscopic currents of the I_ANO6_ of ANO6 variants V1 (left panel), V2 (middle panel), and V5 (right panel) in an inside-out patch clamp from ANO6 variant-transfected HEK293T cells. The patches were exposed for 40–60 s to Ca^2+^ concentrations of 300 nM, 1 μM, and 3 μM at a holding potential of +60 mV. (**b**) Statistics of the peak I_ANO6_ with different calcium concentrations at 27 °C (open-square) and 37 °C (solid-square); graphs displayed at the bottom represent the expansion of the boxed parts of the upper graphs. Data are presented as the means ± SEM (n = 5 for V1, left; n = 6 for V2, middle; and n = 7 for V5, right). NS indicates not significant *P < 0.05, ***P < 0.001 comparing current values at 37 °C and 27 °C.

We also compared the effects of a serial increase in [Ca^2+^]_i_ (0.3, 1, and 3 µM) on I_ANO6_ under inside-out patch clamp conditions with symmetrical NMDG-Cl solution (I_ANO6,i-o_). After membrane excision, I_ANO6,i-o_ could be immediately activated by raising the [Ca^2+^]_i_ (Fig. 7). As shown in Fig. 7a,b, although we increased the bath solution temperature to 37 °C, all variants of ANO6 (V1, V2, and V5) were not activated by a submicromolar Ca^2+^ concentration (300 nM), which differed from the results of whole-cell patch recordings. However, at 1 µM free Ca^2+^, V2 and V5 were significantly activated upon a temperature increase to 37 °C, whereas V1 activation did not reach significance. At 37 °C, V2 and V5 showed approximately 25-fold increases versus those activated at room temperature, even though the current values were numerically small (V2 and V5 generated current values of 1.3 ± 0.8 and 2.6 ± 1.2 pA at room temperature and 12.4 ± 4.9 and 32.2 ± 10.4 pA at 37 °C, respectively). In addition, the amplitude of I_ANO6,i-o_ with 3 µM of [Ca^2+^]_i_ was significantly increased by raising the temperature by approximately 7-, 3-, and 4-fold for V1, V2, and V5, respectively (Fig. 7b). However, I_ANO6,w-o,_ was less sensitive to the temperature increase, as V1 showed almost no change in peak current and V2 and V5 showed approximately 1.7- and 1.8-fold increases in peak current, respectively (Fig. 6e).

### PLA2-independent channel activation of ANO6 variants

**Figure 8:**
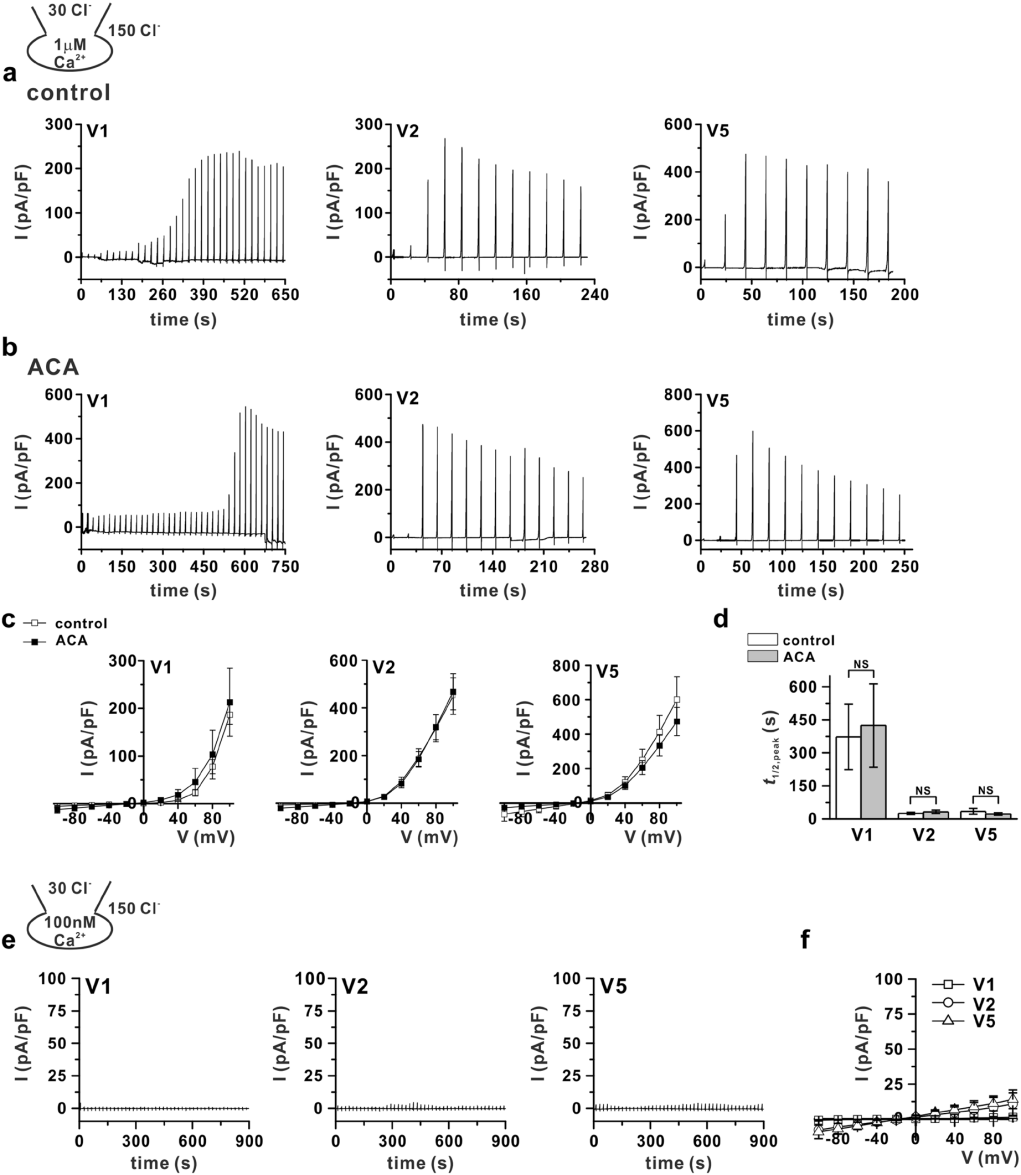
PLA2-independent activation of ANO6 variants 1, 2, and 5. (**a**,**b**) Representative current traces of I_ANO6_ generated from the three variants (V1, left panel; V2, middle panel; and V5, right panel) with 1 μM free calcium and 30 mM Cl^−^ in pipette solution at 37 °C, (**a**) control conditions and (**b**) ACA (20 μM)-treated conditions, in ANO6 variant-expressed HEK293T cells. (**c**) The peak current (I) - voltage (V) relation curve obtained from each variant detected under control conditions (open-square) and ACA-treated conditions (solid-square). (**d**) Summary bar graph of *t*_1/2,peak_ of I_ANO6_ of each variant estimated from the trace chart. Data represent the means ± SEM (n = 7 for V1 and V2, n = 6 for V5). NS indicates not significant, compared to the control. (**e**) Representative trace of the I_ANO6_ of V1 (left-panel), V2 (middle-panel), and V5 (right-panel) with 100 nM free calcium and 30 mM Cl^−^ in pipette solution at 37 °C. (**f**) Corresponding current (I)–voltage (V) relationship curve obtained from the peak currents of V1- square, V2 - circle, V5 - up-triangle. Data represent the means ± SEM (n = 4 for V1, V2, and V5).

Recently, Schreiber *et al*. reported that ANO6 can be partially activated at 37 °C even under intracellular Ca^2+^-free conditions, which might be due to the spontaneous activity of PLA2^29^. However, activation of PLA2 could not activate I_ANO6_ at a high intracellular Cl^−^ concentration, which inhibits I_ANO6_^30^. Therefore, we performed additional experiments to determine whether PLA2 affects the temperature sensitivity of ANO6 variants. We conducted the experiment under two conditions: 150 and 30 mM intracellular Cl^−^ conditions. As shown in Fig. 8a–d, in 30 mM Cl^−^ cytosolic conditions at 37 °C with 1 μM [Ca^2+^]_i_, the *t*_1/2,peak_ of I_ANO6_ variants were not affected by treatment with the PLA2 inhibitor (ACA 20 μM). Moreover, the ANO6 variants could not be activated, even at low cytosolic Cl^−^ at 37 °C with resting intracellular Ca^2+^ (100 nM) (Fig. 8e,f). Furthermore, in the 150 mM Cl^−^ condition, treatment with purified PLA2 (0.5 unit/ml), contained in a pipette solution, or 50 μM NEM (PLA2 activator) could not activate the ANO6 variants at 27 °C with 1 μM [Ca^2+^]_i_. Conversely, at 37 °C, treating I_ANO6_ with the PLA2 inhibitor (ACA 20 μM and MAFP 5 μM) did not affect the *t*_1/2,peak_ (Supplementary Fig. S4).

### Ca^2+^-dependence of the lipid scramblase activity of ANO6

**Figure 9:**
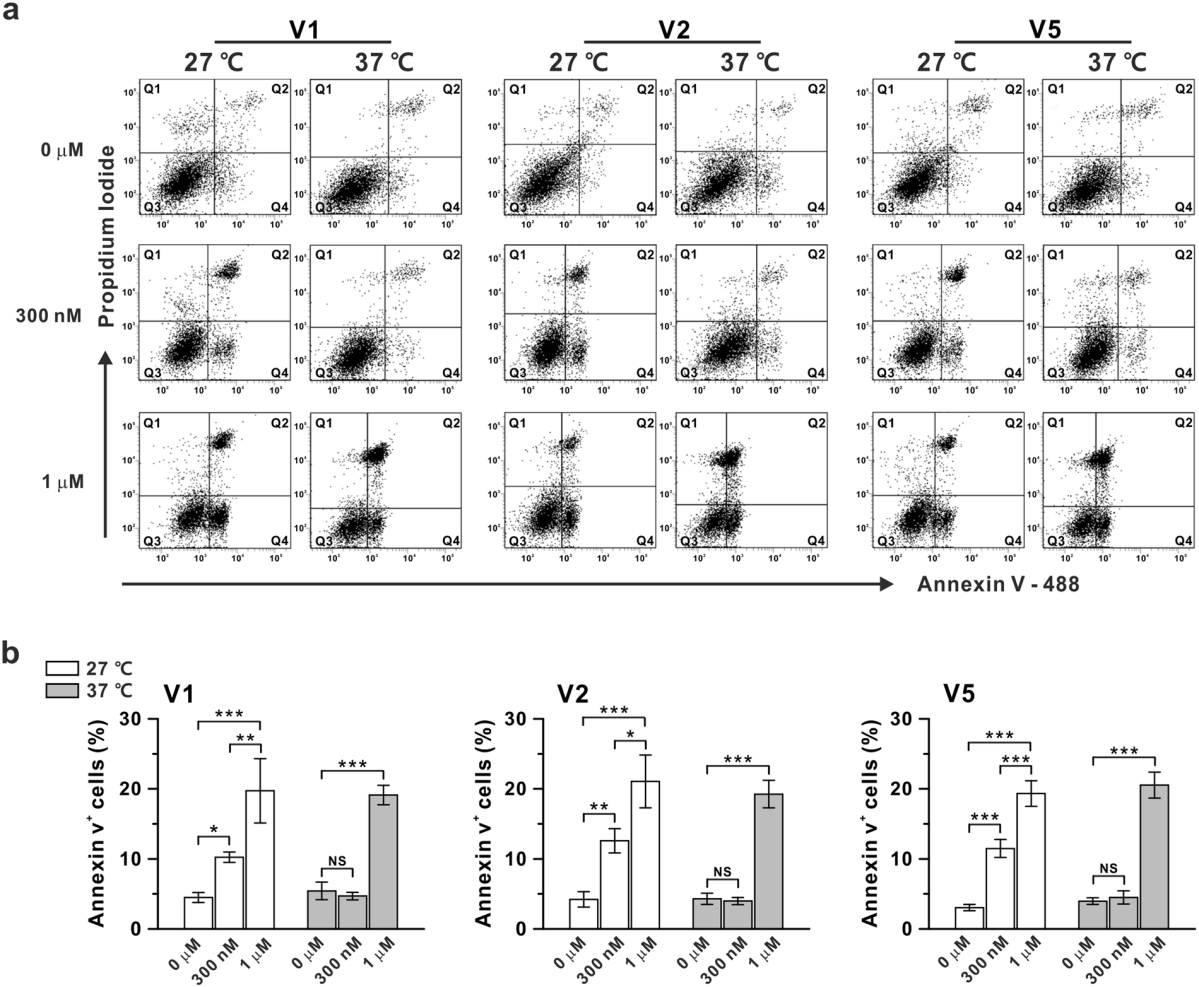
Phosphatidylserine scramblase activity of ANO6 variants V1, V2, and V5 with different [Ca^2+^]_i_. (**a**) Annexin V-binding (X-axis) and PI-binding (Y-axis) cell counts in ANO6 variant-transfected HEK293T cells from flow cytometry. Cells were treated with 10 μM of ionomycin for 15 min at 27 °C or 37 °C. The incubation solution was NT solution containing free calcium concentrations of 0 μM, 100 μM, or 200 μM for the 27 °C condition and 0 μM, 30 μM, or 100 μM for the 37 °C condition. (**b**) Summary bar graph of the response of Annexin V-positive cells [%, percentage of total cell counts (Q4-region)] to ionomycin treatment for each variant (V1- left panel, V2- middle panel, and V5- right panel). Data are presented as the means ± SEM (n = 5–10). NS means not significant; *P < 0.05, **P < 0.01, ***P < 0.001 compared to the control.

The notable increase in the Ca^2+^ sensitivity of I_ANO6_ at 37 °C raised a question whether the scramblase activities of the ANO6 variants are similarly enhanced under physiological temperature. Thus, we conducted a scramblase assay in the V1-, V2-, and V5-overexpressing HEK293T cells. To determine the extracellular free calcium concentration corresponding to the approximate [Ca^2+^]_i_ in intact cells, we first measured [Ca^2+^]_i_ in ANO6-overexpressing HEK293T cells treated with 10 μM ionomycin using a Fura-2 acetoxymethyl ester (Fura-2 AM) probe^31^. Then, for the scramblase assay, we estimated the required free calcium concentration of normal tyrode (NT) solution that induces [Ca^2+^]_i_ close to that attained by the whole-cell patch recordings (300 nM and 1 μM). The free calcium concentrations of NT solution for 300 nM and 1 μM [Ca^2+^]_i_ were 100 μM and 200 µM with ionomycin at 27 °C, respectively, and 30 μM and 100 μM at 37 °C, respectively (Supplementary Fig. S5). The cells were treated with 10 μM ionomycin and the appropriate concentrations of free Ca^2+^ in NT solution for 15 min at 27 °C or 37 °C. To avoid the binding of Annexin V to intracellular phosphatidylserine (PS) resulting from membrane damage, we compiled the statistics using only Annexin V-positive/propidium iodide (PI)-negative cells (Q4-region) (Fig. 9a). The adjusted vertical and horizontal lines for the 4-quadrant windows of Annexin V-/PI-binding were based on the Annexin V and PI single staining performed along with each experiment, respectively (Supplementary Fig. S6a). As shown in Fig. 9b, the scramblase activity of ANO6 was significantly increased within 15 min with 1 μM [Ca^2+^]_i_ at 37 °C, whereas no scramblase activity was detected under 300 nM [Ca^2+^]_i_. Conversely, under a lower temperature condition (27 °C), the scramblase activity of ANO6 was induced with both 300 nM and 1 μM [Ca^2+^]_i_, the concentrations at which channel activation of ANO6 at 27 °C could not be induced.

## Discussion

Despite the expression of ANO6 (TMEM16F) in various cell types^26,32^, the physiological roles of ANO6 have been identified in only limited types of cells such as immunocytes, platelets, and osteoblasts^9,13–15^, all of which are thought to be related to its PS scramblase activity^33–35^. Conversely, owing to its low calcium channel sensitivity, the potential of ANO6 to function as an ion channel under physiological conditions remains controversial. The present results provide evidence that a prolonged physiological increase in [Ca^2+^]_i_ indeed activates ionic currents through ANO6 (I_ANO6_) above 37 °C, and there is a differential temperature-dependence of ANO6 variants. Intriguingly, the I_ANO6_ of V2 and V5 was detected without significant induction of the scramblase activities of ANO6 variants with 300 nM [Ca^2+^]_i_ at 37 °C. Although a recent report by Schreiber *et al*. showed that ANO1 and ANO6 (V1) could be activated at resting Ca^2+^ concentrations at 37 °C^29^, our study shows wider ranges of Ca^2+^-sensitivity depending on the variants of ANO6.

The physiological temperature could dramatically lower the [Ca^2+^]_i_ required for I_ANO6_ activation, with V2 and V5 being activated even by 300 nM [Ca^2+^]_i_ at 37 °C. Moreover, a temperature increase to 42 °C further increased the calcium sensitivity of ANO6, such that V1 could be activated by 300 nM [Ca^2+^]_i_. At 42 °C, all variants could be slightly activated even with resting Ca^2+^ concentrations of 100 nM, while there was no activation with Ca^2+^-free pipette solution even at the higher temperature of 42 °C. The temperature-dependence of I_ANO6_ was reversible (Fig. 3), indicating that the dramatic changes in the threshold [Ca^2+^]_i_ were not due to unspecific leakiness of the patch clamp conditions.

Another remarkable finding was the acceleration of I_ANO6_ development, i.e. decreased *t*_1/2,*peak*_ at the higher temperature. Although a recent study of ANO6 variants showed different kinetics of activation with 20 μM [Ca^2+^]_i_^26^, at room temperature, the development of I_ANO6_ requires tens of seconds for the detectable development of I_ANO6_ and several minutes for steady-state activation even with a relatively high [Ca^2+^]_i_^20–22^. In the present study, we compared the changes in *t*_1/2,peak_ with 3 μM [Ca^2+^]_i_, which showed shortening of the *t*_1/2,peak_ by approximately 50% in all three variants at 37 °C. It was notable that, in the inside-out condition, I_ANO6,i-o_ showed no significant time delay of activation irrespective of temperature, while the calcium sensitivity was decreased (Fig. 7. *t*_1/2,*peak*_: 4 ± 2 s for all variants at 3 μM calcium-free conditions at both 27 °C and 37 °C). Comparison of I_ANO6,i-o_ with the whole-cell current (I_ANO6_) revealed that unknown cytosolic factor(s) might underlie the unique slow activation (see below).

In our study, irrespective of the variant types, the outwardly rectified I/V curves of ANO6 were not altered by temperature even with 100 µM [Ca^2+^]_i_. Such a property was markedly different from that of ANO1, which shows linearization of the I/V curve at higher [Ca^2+^]_i_. Surprisingly, Schreiber *et al*. showed that I_ANO6_ could produce a linear I/V curve, i.e. large inward current, at 37 °C with resting [Ca^2+^]_i_^29^. The same group (Ouisigsawat *et al*.) also reported that a sudden increase in intracellular Ca^2+^ concentration by ionophore leads to a rapid activation of I_ANO6_ and development of a linear I/V curve^13^. However, in our experience, I_ANO6_ always shows outward rectification even at a very high [Ca^2+^]_i_ (>50 μM), which has been confirmed by other research groups^20–22,26,36^. Shimizu *et al*. also reported that the ionomycin-induced [Ca^2+^]_i_ increase still showed delayed activation of I_ANO6_ and a persistent outwardly rectifying I-V relationship^21^. As shown by Stolz *et al*., hANO6 overexpressed in *Xenopus* oocytes could not be activated by ionophore (A23187)-induced calcium increase or calcium influx through ATP-activated P2X7 receptors. This may indicate that ANO6 activation requiures long-term exposure (delayed activation) to high Ca^2+^ levels and that these high Ca^2+^ levels may not be achieved in *Xenopus* oocytes through the application of A23187 or the activation of hP2X7R by ATP^37^.

Despite the marked changes in threshold [Ca^2+^]_i_ for activating I_ANO6_, the peak outward current amplitudes were not significantly increased by raising the temperature in all three variants at 3 μM [Ca^2+^]_i_. The delta amplitude of I_ANO6_ detected between 27 °C and 37 °C only increased by approximately 2-fold compared to those detected at room temperature in V2 and V5, whereas V1 showed the same level of activation currents between room temperature (27 °C) and 37 °C. The results differ from the higher temperature sensitivity of ANO1, a type of heat-sensing ion channel, in terms of the current amplitude changes. It has been reported that the Q_10_ (the 10 °C temperature coefficient) value of ANO1 is approximately 19.4^5^. Nevertheless, the shortened *t*_1/2,peak_ as well as submicromolar [Ca^2+^]_i_ threshold in the ANO6 variants at physiological temperatures (37 °C–42 °C) are plausible conditions in specific physiological contexts, such as under GPCR stimulations for numerous Ca^2+^-sensitive processes^38^, implying the likely electrical roles of ANO6 under physiological conditions.

Abolishment of the latency period for ANO6 activation after membrane excision was described in our previous study^22^, and confirmed with all three variants (Fig. 7). The dramatic difference in the activation kinetics between I_ANO6_ and I_ANO6,i-o_ suggests that unknown cytosolic factor(s) potently inhibit the activation of ANO6, even with a high [Ca^2+^]_i_ combined with the physiological temperature. The candidate intrinsic factors include ATP, phosphorylation states of signalling molecules, cytoskeleton- or calmodulin-dependent signals, and alterations in membrane phospholipids, among others. PLA2 is reported to activate ANO6 only in low cytosolic chloride conditions^29,30^. However, in our study, we conclude that the temperature-dependent calcium sensitivity and acceleration of ANO6 variant activation were not due to PLA2 both in symmetrical and low cytosolic chloride conditions (Figs 8 and S3.). Recently, we revealed that actin cytoskeleton disruption accelerates both delay time and channel inactivation of ANO6^39^. However, more studies are needed to clarify the relationship between temperature changes and actin cytoskeleton rearrangement. Because more research is needed to verify this hypothesis, we have not yet conducted a rigorous investigation to specify the inhibitory factors of ANO6, which will be addressed in our future studies.

Notably, abolishment of the submicromolar [Ca^2+^]_i_ activation of I_ANO6,i-o_ in the excised patches at 37 °C indicated that unknown cytosolic factors are also likely required for the critical temperature-dependence of ANO6. Although the size of I_ANO6,i-o_ with 3 μM was increased at 37 °C, such changes might reflect the natural thermodynamic energy state of the temperature increase rather than the notable physiological temperature sensitivity. Although, more evidence is required to prove this phenomenon, it might be caused by the structural difference between ANO6 variants that underlie their distinguishable biophysical properties, including their Ca^2+^ sensitivity and *t*_1/2,peak_. Consistent with this, the results of Scudieri *et al*.^26^ and our study indicate that the Ca^2+^ sensitivity and *t*_1/2,peak_ differ among ANO6 variants. Generally, V2 and V5 are more sensitive to [Ca^2+^]_i_ and exhibit relatively faster *t*_1/2,peak_ than V1 at all temperature ranges (Figs 4 and 6). However, in excised patch-clamp conditions, the difference in Ca^2+^ sensitivity and *t*_1/2,peak_ between each variant disappears (Fig. 7).

The altered domain structures in the N- and C-termini of the variants have been schematically compared^26^. According to the sequence database, V2 contains only 5 amino acids in contrast to the 23 amino acids in V1 at the initial part of the N-terminus, whereas V5 has an additional 21 amino acids inserted following the first 23 amino acids of V1. Notably, the segment at the first 23 amino acids contains a stretch of eight consecutive acidic amino acid residues (EEEEDDDD). A similar case can be observed in the mammalian Na+/H+ exchanger isoform 1 (NHE1), which has seven consecutive acidic residues in the distal region of the cytosolic tail^40^. This site is critical for the maintenance of NHE1 activity and calmodulin binding. Thus, V2 and V5 may be weaker or less affected by the specific regulatory factor than V1. In many cases, as with NHE1, consecutive negative acidic amino acids are often used to regulate the activity of ion channels in the cytoplasm. Therefore, it is necessary to identify the role of this domain in future studies through mutation analyses. As a whole, these findings suggest that the cytosolic initial amino acids of the N-terminus play an important role for sensing Ca^2+^ and determining the delayed time for activation. Nevertheless, the exact mechanism of ANO6 activation and the reason for the observed delayed activation remain unknown.

ANO6 is also well recognized as a membrane phospholipid scramblase^34^. A recent elaborate study demonstrated that the ionic flow through ANO6 owes to a leak current associated with lipid transport via scramblase activity. Although the experimental results were obtained with 200 μM [Ca^2+^]_i_, this might imply that the ions and phospholipids share the same pathway^41^. Additionally, the most recent research established that ion conductance and lipid scrambling, which require a high calcium concentration with EC_50_ of 8 μM for activation, were activated by the same Ca^2+^ activation mechanisms^42^. However, the temperature-dependence of lipid scramblase activity appears inconsistent; temperature reductions did not slow lipid transport^43^, while another report established that ANO6-induced PS exposure was sufficiently rapid at 25 °C (RT), although the rate was faster at 37 °C^44^. However, in our study, the channel and scramblase activities of ANO6 seemed to be separately induced as the mechanism of the temperature-dependent increase in the calcium sensitivity of the ANO6 channel was not observed for the scramblase activity. When comparing the 27 °C condition and the physiological temperature (37 °C) condition, the calcium sensitivity was higher than the scramblase activity at 27 °C, since the PS exposure occurred at 300 nM [Ca^2+^]_i_ at 27 °C, whereas at 37 °C, scramblase activity was activated with 1 μM [Ca^2+^]_i_ but not with submicromolar [Ca^2+^]_i_.

**Figure 10:**
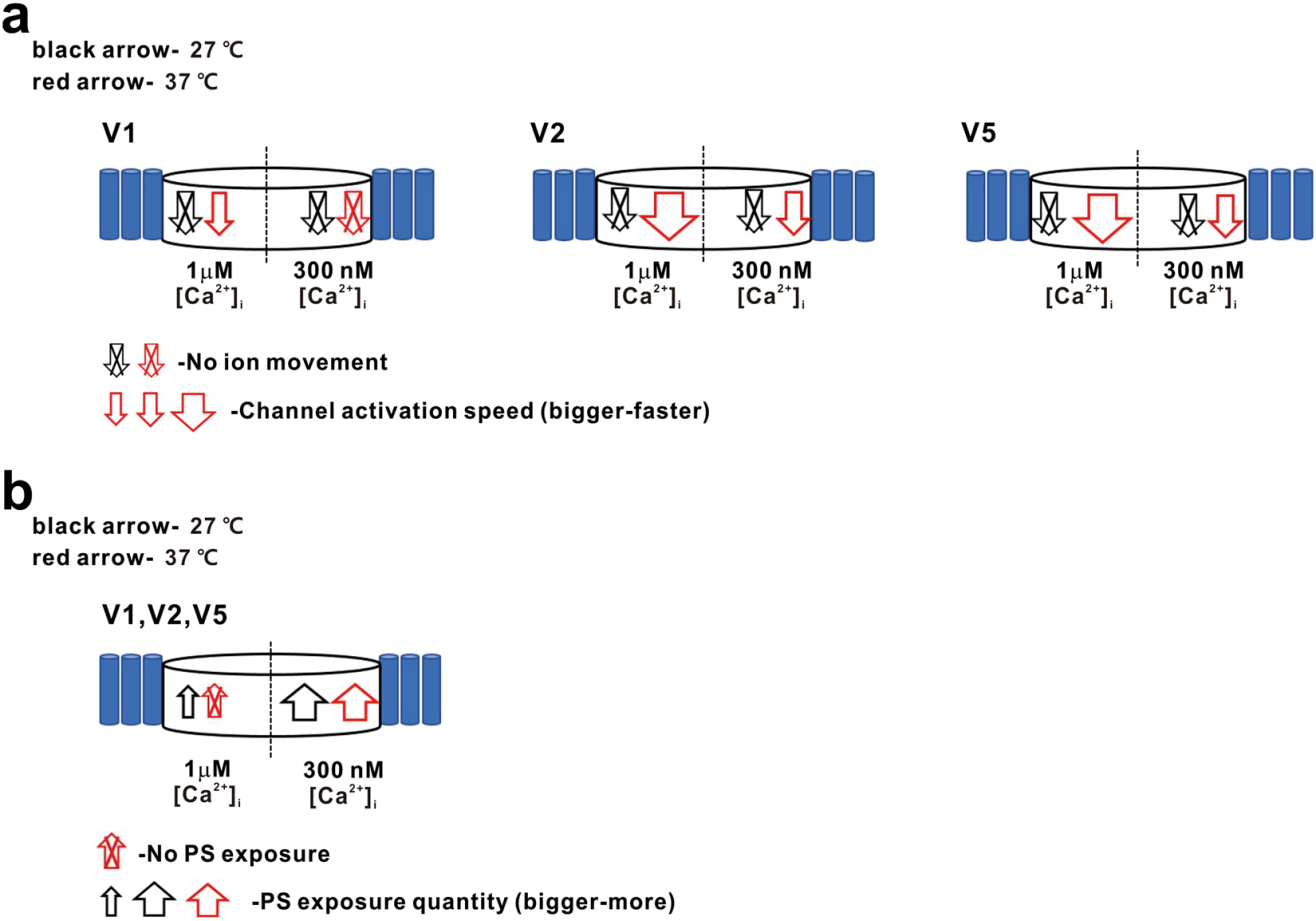
Summary model for temperature-dependent channel activity and scramblase activity of ANO6 variants. (**a**) Calcium-dependent channel activity and (**b**), PS exposure response to raised temperature. Black arrow - 27 °C, Red arrow - 37 °C, Arrow with cross bar - no movement of ion or PS, Open arrow - ion movement or PS movement.

Although the results require careful interpretation, the scramblase and channel activities of ANO6 might utilize discriminable molecular mechanisms *in vivo* under physiological conditions, further supporting the physiological function of ANO6 as an ion channel (Fig. 10). However, the ionic flow through ANO6 might be more sensitively detected using the voltage clamp technique with a persistent voltage gradient. In addition, one has to consider that the PS exposure levels detected in the fluorescence-activated cell sorting (FACS) analysis reflect summed effects of the scramblase and the intrinsic flippase activity reversing the PS to the inner leaflet^33^. In this respect, the relatively low scramblase activity at the physiological temperature (37 °C) at submicromolar [Ca^2+^]_i_ might have resulted from the compensatory effects of flippase, which may be stronger at 37 °C than at room temperature (27 °C).

In summary, we provide the first demonstration that the ANO6 isoforms V2 and V5 can be activated within a submicromolar range of cytosolic free calcium at 37 °C, and that V1 can be activated by 1 μM [Ca^2+^]_i_ with a short *t*_1/2,peak_. At 42 °C, all ANO6 variants show discernible activity even with 100 nM [Ca^2+^]_i_. Thus, we provide an experimental foundation for the functional exploration of ANO6 as an ion channel. The temperature-dependent calcium sensitivity and accelerated activation of ANO6 require additional detailed study to fully understand their precise mechanisms. Interestingly, even at physiological temperatures, the phospholipid scramblase activity of ANO6 seems to require higher calcium concentrations (1 μM) than the ionic current activation. Furthermore, in contrast to the Ca^2+^-dependence of the ionic currents, the scramblase activities of V1, V2, and V5 showed similar patterns. We cautiously suggest that the channel function of ANO6 acts separately from the scramblase activity. Further quantitative analysis of ANO6 variant expression would be necessary for elucidating their physiological roles in different tissues expressing ANO6.

## Methods

### Cell culture

HEK293T cells (American Type Culture Collection, Manassas, VA, USA) were cultured in high-glucose Dulbecco’s modified Eagle’s medium (DMEM-HG; Thermo Fisher Scientific, Waltham, MA, USA) supplemented with 10% foetal bovine serum (FBS; Thermo Fisher Scientific). Cells were maintained in a 37 °C humidified incubator at 20% O_2_/5% CO_2_, and were subcultured every 2 or 3 days. HEK293T cells were cultured in 35-mm dishes and 75-T flasks for electrophysiology, calcium measurements, and FACS analysis.

### Plasmids and transfection

The mammalian expression plasmid used for expressing the human ANO6 (hANO6) V1 transcript was described previously^22^. The cDNAs of hANO6 V2, V3, and V5 transcripts were purchased from GeneCopoeia^TM^ (Rockville, MD, USA). The coding regions of hANO6 V1 (GenBank accession no. NM_001025356.2), V2 (NM_001142678.1), V3 (NM_001142679.1), and V5 (NM_001204803.1) were subcloned into the mammalian expression vector pcDNA 3.1(+) (Thermo Fisher Scientific) with a Kozak consensus sequence (GCC ACC) immediately upstream of the initiation codon using polymerase chain reaction amplification. HEK293T cells were maintained in DMEM-HG supplemented with 10% FBS and 1% penicillin and streptomycin (Thermo Fisher Scientific). The plasmids were transiently transfected to HEK293T cells using Turbofect transfection reagent (Thermo Fisher Scientific) according to the manufacturer’s instructions. For electrophysiological experiments, HEK293T cells (cultured in 35-mm dishes) were co-transfected with 0.9 μg of the hANO6 variant plasmids along with 0.1 μg of the GFP expression plasmid to visualise the transfected cells. For western blotting, calcium measurements, and FACS analysis, HEK293T cells (grown in 75-T flasks) were transfected with 4.5 μg of each ANO6 variant plasmid. Experiments were performed within 24–36 h after transfection.

### Electrophysiology

Whole-cell and inside-out patch clamp techniques were applied for measuring the channel activities of ANO6 variant-transfected HEK293T cells. Cells were transferred to a bath mounted on the stage of an IX-50 inverted microscope (Olympus, Osaka, Japan) equipped with a light source set to green fluorescence excitation wavelengths. The patch-clamp experiment was performed at 27 °C or 37 °C. Each temperature was maintained by a circulating water bath and was continuously measured in a bath chamber to be maintained at exactly 27 °C or 37 °C (KeumSung Scientific, Seoul, Korea). Microglass pipettes (World Precision Instruments, Sarasota, FL, USA) were fabricated using a PP-830 single-stage glass microelectrode puller (Narishige, Tokyo, Japan), with a resistance of 2.5–3.5 MΩ and 5–6 MΩ for whole-cell and inside-out patch recordings, respectively, using an MF-830 microforge (Narishige, Tokyo, Japan). Currents were recorded using an Axopatch 200B amplifier and Digidata 1332 A interface, digitised at 10 kHz and low pass-filtered at 5 kHz by pClamp software 10.3 (Molecular Devices, Sunnyvale, CA, USA). The series resistance and junction potential were compensated by an offset circuit in Axopatch 200B. All voltage and current trace data were analysed using Clampfit 10.3 and Origin 8.0 software (Microcal, Northampton, MA, USA). The detailed stimulation voltage protocols are described in the relevant figure legends.

### Solutions

The whole-cell patch clamp analysis of ANO6 was conducted using a basal extracellular solution containing 146 mM NMDG-Cl, 1 mM CaCl_2_, 1 mM MgCl_2_, 10 mM HEPES, and 5 mM glucose (adjusted to pH 7.4 with NMDG-OH). The basal pipette solution contained 10 mM EGTA, 5 mM HEPES, 0.5 mM MgCl_2_, and 1 mM Mg-ATP; an appropriate amount of CaCl_2_ was added to the pipette solution to obtain a Ca^2+^ concentration of 0.1, 0.3, 1, 3, and 100 µM for the 27 °C, 37 °C, and 42 °C experimental condition (adjusted to pH 7.2 with NMDG-OH). WEBMAX-C software (C. Patton, Stanford University, www.stanford.edu/~cpatton/maxc.html) was used to calculate the accurate amount of CaCl_2_ for each free Ca^2+^ concentration at 27 °C, 37 °C, and 42 °C. Because Ca^2+^ chelation by EGTA is weak when the free Ca^2+^ concentration is 100 µM, 10 mM HEDTA was used. Given that two Cl^−^ ions bind to one Ca^2+^ ion, appropriate amounts of NMDG-Cl were added to the basal pipette solution to adjust the Cl^−^ concentration to 150 mM.

For the inside-out patch clamp experiment, the basal pipette solution contained 150 mM NMDG-Cl, 1 mM EGTA, and 5 mM HEPES (pH 7.4). The bath solution for the intracellular side of the patch contained 150 mM NMDG-Cl, 1 mM EGTA, and 5 mM HEPES (pH 7.2). To obtain different concentrations of free Ca^2+^, 10 mM EGTA or HEDTA was added to the bath solution, and an appropriate amount of CaCl_2_ was added to change the free Ca^2+^ concentration as described above.

### Cell surface biotinylation and immunoblotting assay

Surface biotinylation and immunoblotting were performed using conventional methods as described previously^45,46^. In brief, transfected HEK293T cells were washed three times with ice-cold phosphate-buffered saline (PBS). The cells were then treated with sulpho-NHS-SS-biotin (Pierce, Rockford, IL, USA) containing buffer for 30 min at 4 °C to biotinylate the plasma membrane proteins. After biotinylation, the cells were washed with quenching buffer to remove the excess biotin and washed twice again with PBS. The cells were harvested and incubated overnight with avidin solution (Ultra-Link Immobilised NeutrAvidin Beads 10%, Pierce) at 4 °C. Avidin-bound complexes were washed three times and the biotinylated proteins were eluted in 2 × sample buffer. The protein samples were suspended in a sodium dodecyl sulphate (SDS) buffer and separated by SDS–polyacrylamide gel electrophoresis. The separated proteins were transferred to a nitrocellulose membrane and blotted with appropriate primary and secondary antibodies. Anti-ANO6 (G-14; Santa Cruz Biotechnology, Santa Cruz, CA, USA) antibody was used as the primary antibody, and horseradish peroxidase-conjugated anti-rabbit IgG (Thermo Fisher Scientific) was used as the secondary antibody. Protein bands were detected by enhanced chemiluminescence (Amersham Biosciences, Buckinghamshire, UK).

### Flow cytometry

hANO6 variant-transfected HEK293T cells were incubated with 10 µM of ionomycin for 15 min at 27 °C or 37 °C. The incubation NT solution contained 3.6 mM KCl, 10 mM HEPES, 10 mM glucose, and 10 mM HEDTA (adjusted to pH 7.4 with NaOH), with a fixed Ca^2+^ concentration of 0, 30, 100, or 200 μM. The cells were then resuspended with 100 μL Annexin V binding buffer (2.5 μL Annexin V-488 and 1 μg·mL^−1^ PI; Invitrogen, Carlsbad, CA, USA) in 5-mL polystyrene round-bottom tubes. The tubes were then gently vortexed and incubated in the dark for 15 min at 4 °C, and 400 μL of the binding buffer was added to each tube before flow cytometry analysis using the FACSCalibur system (BD Bioscience, San Jose, CA, USA). To avoid the influence of ionomycin on the cells, the treated cells were maintained at 4 °C for all processes until the FACS analysis.

### Statistical analysis

The results are expressed as the means ± SEM. Statistical analysis was performed by one-way ANOVA with post-hoc Tukey’s HSD tests and two-way ANOVA with Bonferroni post-tests for multiple comparisons. *P* < 0.05 was considered statistically significant.

## Supplementary information

Supplementary Information

Supplementary Material
