# Supplementary material for: Temperature-dependent increase in the calcium sensitivity and acceleration of activation of ANO6 chloride channel variants
Source: Sci Rep. 2019 Apr 30;9:6706. doi: 10.1038/s41598-019-43162-1 (PMC6491614; doi:10.1038/s41598-019-43162-1)

Temperature-dependent increase in the calcium sensitivity and acceleration of activation of ANO6 chloride channel variants

Haiyue Lin[1,#], Ikhyun Jun[2,3,#], Joo Han Woo[4,5], Min Goo Lee[3], Sung Joon Kim[1,*] & Joo Hyun Nam[4,5,*]

Supplementary Information

Figure S1. Figure showing uncropped western blots from Fig. 1a.

Surface biotinylation (upper) and whole cell lysate western blotting (middle-ANO6 blotting, bottom-aldolase blotting) uncropped membrane images.

Figure S2. I_{ANO6}-V1 with 300 nM $[Ca^{2+}]_i$ at 42°C.

(**a**) Representative current trace of I_{ANO6} generated from V1-expressed HEK293T cells with 300 nM $[Ca^{2+}]_i$ at 42°C. The relative I-V relation curve for initial current (1) is presented in the right upper panel. (**b**) and (**c**) Mean current (I)-voltage (V) relationship curves obtained from initial (1) and peak (2) currents of the whole-cell patch clamp with 300 nM $[Ca^{2+}]_i$ at 42°C, respectively (n = 5).

Figure S3. I_{ANO6} of variants 1, 2 and 5 detected under calcium-free condition at 42°C

(**a**) Representative trace of the I_{ANO6} of V1 (left-panel), V2 (middle-panel), and V5 (right-panel), in the presence of 0 $[Ca^{2+}]_i$, expressed in HEK293T cells. The pulse protocol is the same as that described in Fig. 2. (**b**) I-V relationship curves obtained from V1 (square), V2 (circle), and V5 (up-triangle) (n = 3 for all variants).

Figure S4. PLA2 effects on I_{ANO6} of variants 1, 2, and 5 under symmetrical Cl^- condition.

(**a, c**) Representative currents trace of I_{ANO6} generated from the three variants (V1, left panel; V2, middle panel; and V5, right panel) at 27°C with 1 μM free calcium under (**a**) 0.5 unit/ml PLA2 in pipette solution and (**c**) NEM (50 μM) treatment conditions in

ANO6-overexpressing HEK293T cells. (**b, d**) Current (I) - voltage (V) relation curve obtained from each variant detected at PLA2 in pipette (**b**) and NEM-treated conditions (**d**), respectively (V1- square, V2 – circle, and V5 - up-triangle). Data represent the means ± SEM (n = 4 for V1, V2, and V5 for all conditions). (**e, f**) Representative currents trace of I_{ANO6} generated from the three variants (V1, left panel; V2, middle panel; and V5, right panel) at 37°C with 1 μM free calcium under ACA (20 μM) treatment (**e**) and MAFP (5 μM) treatment (**f**) conditions, respectively, in ANO6-transfected HEK293T cells. (**g**) Summary bar graph of $t_{1/2,\ peak}$ of I_{ANO6} of each variant estimated from the trace chart of the control (Figure 2b) under ACA and MAFP treatment conditions. Data represent the means ± SEM (n = 5 for V1, V2, and V5 at all conditions). NS indicates not significant, compared to the control.

Figure S5. Intracellular calcium levels induced by ionomycin

(**a**) Representative trace of the intracellular calcium concentration induced in ANO6 (V1, V2, and V5)-expressing HEK293T cells with 10 μM ionomycin. The intracellular calcium levels were measured in NT solution containing free calcium concentrations of 100 μM and 200 μM at 27°C (right), and 30 μM (middle) and 100 μM (left) at 37°C using Fura 2-AM probe. At 27°C, free calcium concentration of NT was changed from 100 μM to 200 μM by adding an appropriate amount of $CaCl_2$ at the time point where the intracellular calcium concentration reached steady state. (**b**) Summary bar graph of the intracellular calcium concentration at 27°C (left) and 37°C (right) (n = 3~4).

Figure S6. PS exposure comparison of V1, V2, and V5.

(**a**) Annexin V-binding (X-axis) and PI-binding (Y-axis) cell counts in ANO6-V1-transfected HEK293T cells from flow cytometry. The correct vertical and horizontal lines for the 4-quadrant windows of Annexin V-/PI-binding (right) were based on the Annexin V (left) and PI (middle) single staining, respectively. (**b**) Summary bar graph of the response of Annexin V-positive cells [%, percentage of total cell counts (Q4-region)] to ionomycin treatment for each variant at 27°C (left) and 37°C (right), estimated from data presented in Figure 9. Data are presented as the means ± SEM (n =

5~10). NS means not significant compared to V1.

Supplementary Figure 1.

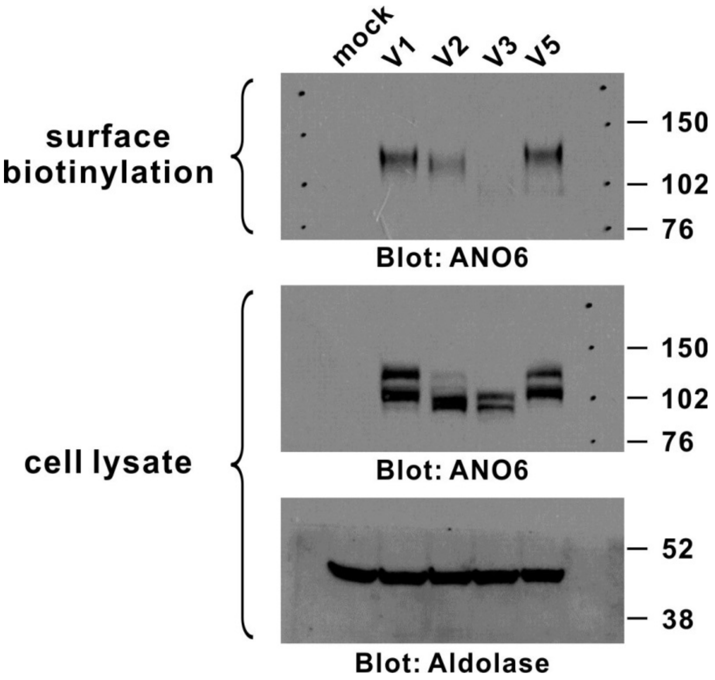

Supplementary Figure 2.

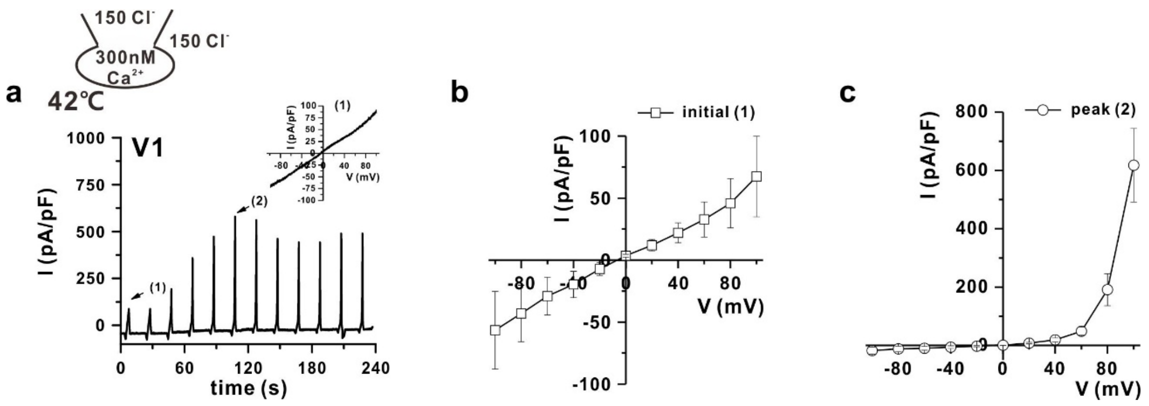

Supplementary Figure 3.

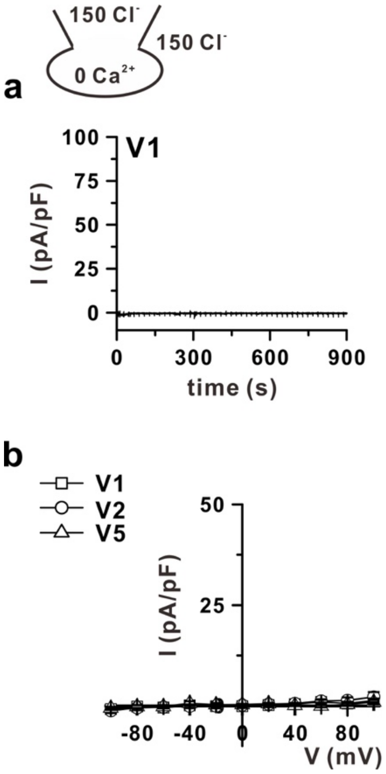

Supplementary Figure 4.

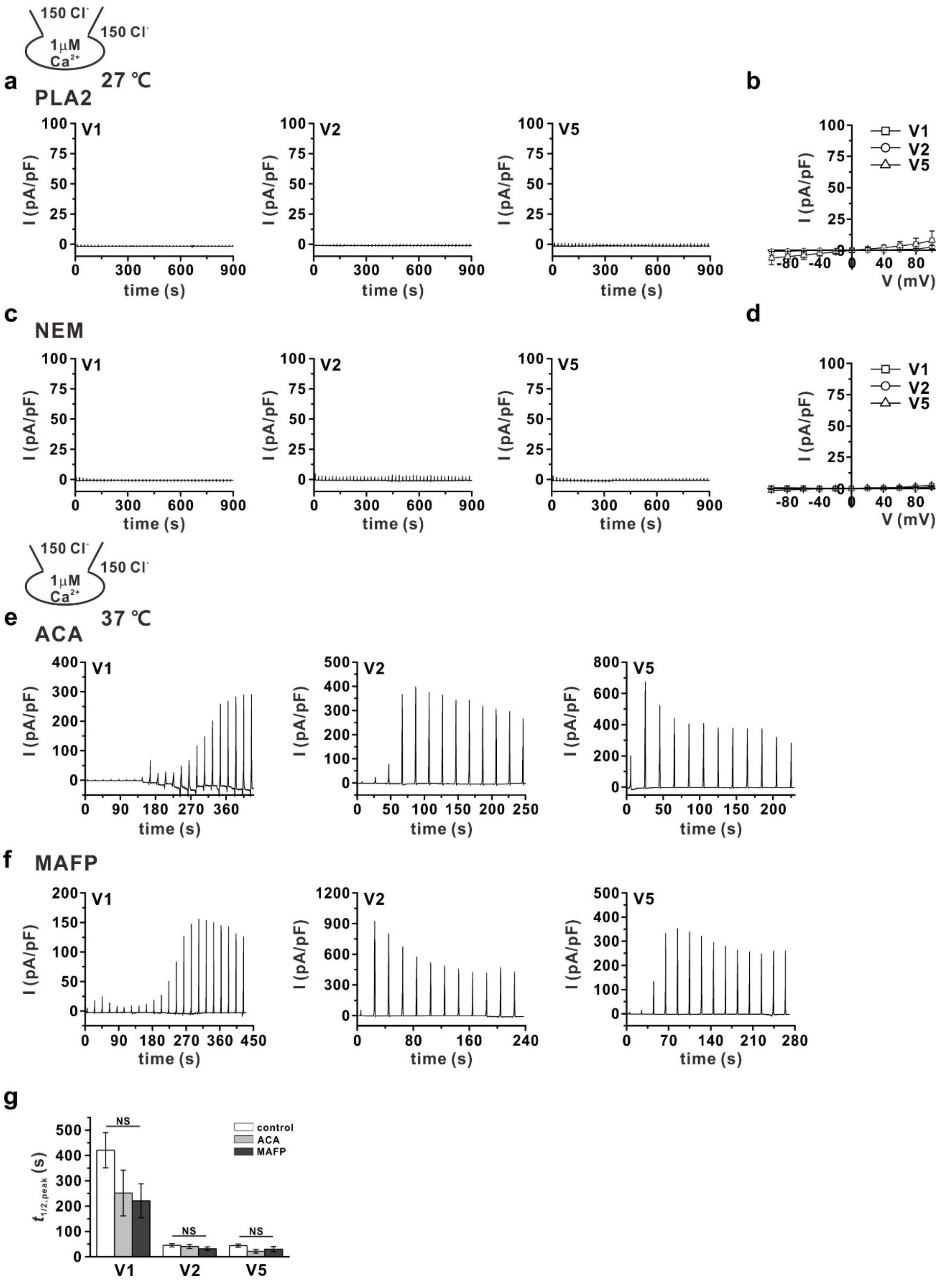

Supplementary Figure 5.

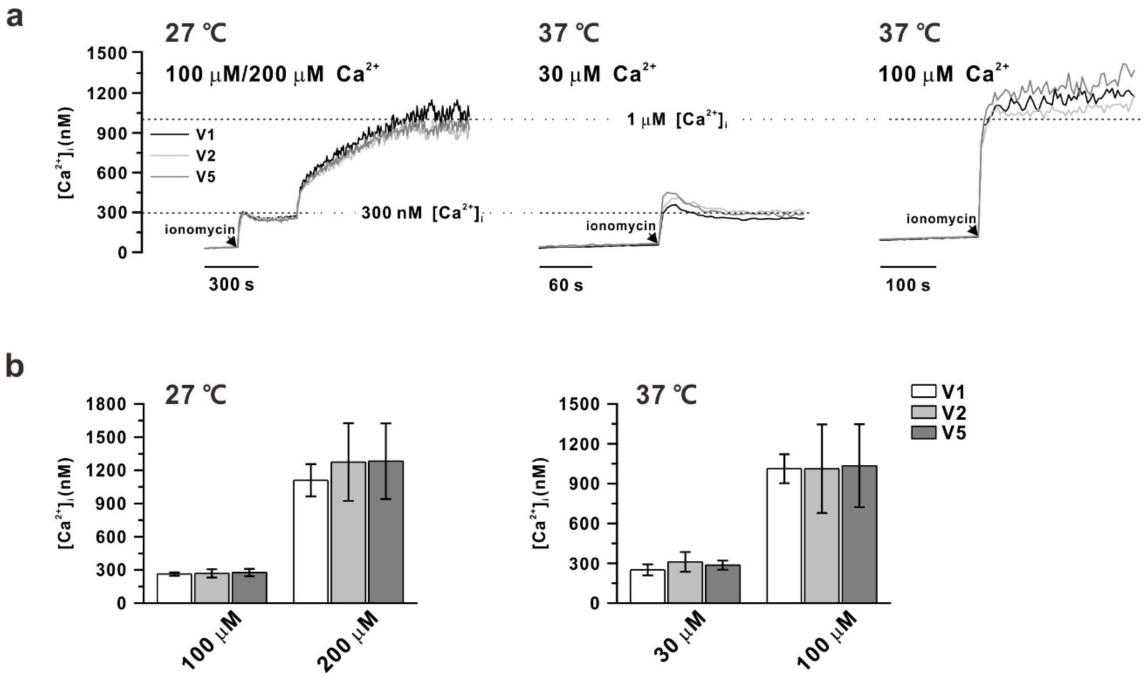

Supplementary Figure 6.

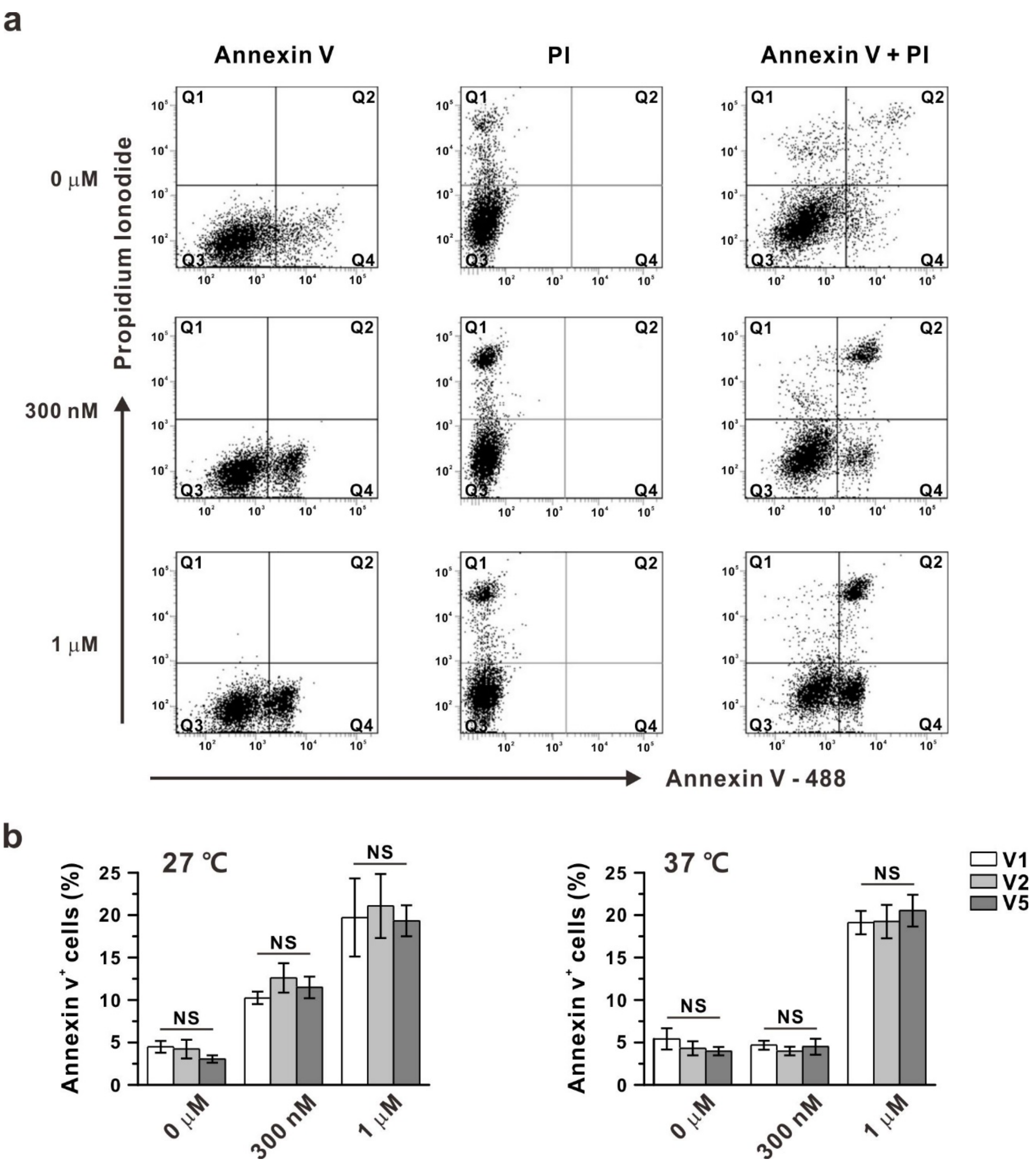

Supplement: Supplementary file 1 — Supplementary Information [file 41598_2019_43162_MOESM1_ESM.pdf]
