# Supplementary material for: Temperature-dependent increase in the calcium sensitivity and acceleration of activation of ANO6 chloride channel variants
Source: Sci Rep. 2019 Apr 30;9:6706. doi: 10.1038/s41598-019-43162-1 (PMC6491614; doi:10.1038/s41598-019-43162-1)
Supplement: Supplementary file 2 — Supplementary Material [file 41598_2019_43162_MOESM2_ESM.pdf]

Temperature-dependent increase in the calcium sensitivity and acceleration of activation of ANO6 chloride channel variants

Haiyue Lin[1,#], Ikhyun Jun[2,3,#], Joo Han Woo[4,5], Min Goo Lee[3], Sung Joon Kim[1,*] & Joo Hyun Nam[4,5,*]

Supplementary Material

Intracellular calcium measurement. $[Ca^{2+}]_i$ was measured using the fluorescent Ca^{2+} indicator, Fura-2 AM (Thermo Fisher Scientific). HEK293T cells were transiently transfected with hANO6 variants (V1, V2, and V5), incubated with NT solution (145 mM NaCl, 10 mM HEPES, 5 mM glucose, 3.6 mM KCl, 1.5 mM $CaCl_2$, and 1 mM $MgCl_2$; adjusted to pH 7.4 with NaOH) containing 2 μM Fura-2 AM for 30 min at 37°C, and then washed twice with NT solution. The cells were then resuspended with NT solution containing fixed Ca^{2+} concentrations of 0, 30, 100, or 200 μM for the 27°C and 37°C experimental conditions. Given that two Cl^- ions bind to one Ca^{2+} ion, an appropriate amount of NaCl was added to the NT solution to adjust the Cl^- concentration to 150 mM. Then, the 10 μM ionomycin-induced calcium increase (fluorescence) was monitored in a quartz microcuvette (1 mL) with stirring in a temperature-controlled (27°C or 37°C) cell holder of a fluorescence spectrophotometer (Photon Technology International, Birmingham, NJ, USA) at excitation wavelengths of 340 nm and 380 nm, and an emission wavelength of 510 nm. At the end of each experiment, an appropriate amount of $CaCl_2$ was added to adjust the final concentration of $CaCl_2$ to 1.3 mM to produce the maximum fluorescence ratio (R_{max}: 340/380 nm). Subsequently, 20 mM EGTA was added to confirm the minimum fluorescence ratio (R_{min}: 340/380 nm). The $[Ca^{2+}]_i$ values were calculated using the equation $[Ca^{2+}]_i = K_d \times b \times (R - R_{min})/(R_{max} - R)$, where K_d is the dissociation constant (224 nM for 37°C, and 252 nM for 27°C)[31] of Fura-2-AM and b is the ratio of the fluorescence excitation intensities of Fura-2 AM at 380 nm under Ca^{2+}-free and Ca^{2+}-saturated conditions.
